# Engaging the older cancer patient; Patient Activation through Counseling, Exercise and Mobilization – Pancreatic, Biliary tract and Lung cancer (PACE-Mobil-PBL) - study protocol of a randomized controlled trial

**DOI:** 10.1186/s12885-018-4835-2

**Published:** 2018-09-27

**Authors:** Marta Kramer Mikkelsen, Cecilia Margareta Lund, Anders Vinther, Anders Tolver, Anne-Mette Ragle, Julia Sidenius Johansen, Inna Chen, Lotte Engell-Noerregaard, Finn Ole Larsen, Bo Zerahn, Dorte Lisbet Nielsen, Mary Jarden

**Affiliations:** 1 Department of Oncology and Hematology, Rigshospitalet, Copenhagen University Hospital, Blegdamsvej 9, 2100 Copenhagen Ø, Denmark; 2 Department of Oncology, Herlev and Gentofte Hospital, Copenhagen University Hospital, Herlev Ringvej 75, 2730 Herlev, Denmark; 3 Department of Rehabilitation, Herlev and Gentofte Hospital, Copenhagen University Hospital, Herlev Ringvej 75, 2730 Herlev, Denmark; 4 QD-Research Unit, Herlev and Gentofte Hospital, Copenhagen University Hospital, Herlev Ringvej 75, 2730 Herlev, Denmark; 5 Data Science Laboratory, Department of Mathematical Sciences, University of Copenhagen, 2100 Copenhagen Ø, Denmark; 6 Department of Clinical Medicine, Faculty of Health and Medical Sciences, University of Copenhagen, 2200 Copenhagen N, Denmark; 7 Department of Clinical Physiology and Nuclear Medicine, Herlev and Gentofte Hospital, Copenhagen University Hospital, Herlev Ringvej 75, 2730 Herlev, Denmark; 8 Department of Public Health, Faculty of Health and Medical Sciences, University of Copenhagen, 1014 Copenhagen K, Denmark; 9 Department of Medicine, Herlev and Gentofte Hospital, Copenhagen University Hospital, Herlev Ringvej 75, Herlev, 2730 Denmark

**Keywords:** Advanced cancer, Biliary tract cancer, Cancer, Counseling, Exercise, Lung cancer, Mobilization, Multimodal intervention, Older, Pancreatic cancer, Physical activity

## Abstract

**Background:**

Several intervention studies have demonstrated that exercise training has beneficial effects among cancer patients. However, older cancer patients are underrepresented in clinical trials, and only few exercise-based studies have focused specifically on older patients with cancer. In particular, research investigating the effects of exercise training among older patients with advanced cancer is lacking. The purpose of the current study is to investigate the effect of a 12-week multimodal and exercise-based intervention among older patients (≥65 years) with advanced pancreatic, biliary tract or lung cancer, who are treated with first-line palliative chemotherapy, immunotherapy or targeted therapy.

**Methods:**

PACE-Mobil-PBL is a two-armed randomized controlled trial. Participants will be randomized 1:1 to an intervention group (*N* = 50) or a control group (*N* = 50). Participants in the intervention group will receive standard oncological treatment and a 12-week multimodal intervention, comprised of: (I) supervised exercise training, twice weekly in the hospital setting, (II) home-based walking with step counts and goal-setting, (III) supportive and motivational nurse-led counseling, and (IV) protein supplement after each supervised training session. Participants in the control group will receive standard oncological treatment. The primary outcome is physical function measured by the 30-s chair stand test. Secondary outcomes include measures of feasibility, activity level, physical capacity and strength, symptom burden, quality of life, toxicity to treatment, dose reductions, inflammatory biomarkers, body weight and composition, hospitalizations and survival. Assessments will be conducted at baseline, and after 6, 12 and 16 weeks.

**Discussion:**

The current study is one of the first to investigate the effect of an exercise-based intervention specifically targeting older patients with advanced cancer. PACE-Mobil-PBL supports the development of health promoting guidelines for older patients with cancer, and the study results will provide new and valuable knowledge in this understudied field.

**Trial registration:**

The study was prospectively registered at ClinicalTrials.gov on January 26, 2018 (ID: NCT03411200).

**Electronic supplementary material:**

The online version of this article (10.1186/s12885-018-4835-2) contains supplementary material, which is available to authorized users.

## Background

Advanced age is the leading risk factor for development of cancer overall, and approximately 60% of all cancers in Europe and in the USA are diagnosed among patients aged 65 years or older [1, 2]. Although the risk of cancer increases with age, and the number of older patients with cancer is expected to rise in the coming years [3, 4], research on older patients with cancer is limited [1, 4]. Aging involves a continuum of changes in function, biological, psychological, and social structures that vary depending on individual differences. In a biological view, aging causes changes in the organism that lead to a decline in organ functions and in physiological reserves [5–8]. Comorbidity, defined as the occurrence of two or more medically diagnosed diseases, increases with age and heightens the risk of disability and mortality [9, 10]. In addition, use of multiple drugs for multiple diseases, also known as polypharmacy, leads to an increased risk of drug interactions, adverse reactions and poor adherence [11].

### Cancer and aging

Aging itself is associated with limitations in physical function and reduced reserve capacity. A diagnosis of cancer and its accompanying treatments can lead to numerous symptoms and side effects, physical disability, psychological distress and increased health care needs [12]. The interplay between age-related and cancer-related declines in health increases vulnerability and risk of development of short- and long-term disabilities [13, 14]. Older patients with cancer have a higher incidence of limitations in activities of daily living (ADL), reduced quality of life (QoL), and a higher prevalence of geriatric syndromes such as depression, falls and osteoporosis compared to older adults without a history of cancer [15, 16]. Research suggests that older patients with cancer benefit from chemotherapy similar to younger patients [17, 18]. However, older patients with cancer are at increased risk of toxicity due to age-related physiological changes [19].

### Sarcopenia and cachexia

Loss of muscle mass and strength is a part of the normal aging process and is referred to as primary sarcopenia [20]. Sarcopenia has multiple contributing factors including aging itself, insufficient nutrition, sedentary lifestyle or bedrest. The term secondary sarcopenia is often used to describe sarcopenia that is caused by other factors such as cancer or other chronic diseases [20]. Cachexia is another depleting syndrome that causes weight loss and muscle wasting due to an underlying disease [21]. The pathophysiology of cancer cachexia includes a complex combination of reduced energy intake and uptake, hormonal alterations, abnormal metabolism and inflammation [21]. Previous studies have identified cachexia and sarcopenia as contributors to impaired physical functioning [22], reduced tolerance to anti-cancer treatment [23, 24], higher symptom burden [25], reduced QoL [25, 26] and increased mortality [24, 27, 28] among patients with cancer.

### Exercise training

Research has shown that exercise training (e.g. aerobic training, resistance training, and balance and flexibility exercises) is safe and beneficial for older people [29–31]. Documented effects include increased muscle mass and strength, functional mobility and ADL function, improved QoL, and reduction of falls and depressive symptoms [29–32]. The effect of exercise training has also been widely investigated in cancer patients [33–35]. A myriad of beneficial effects of exercise training have been documented, including reduced severity of cancer and treatment-related symptoms and side effects, as well as improved aerobic capacity, muscle strength, physical functioning and QoL [33–36]. However, older patients are underrepresented in clinical trials, and only few exercise-based intervention studies have focused specifically on older patients with cancer [37]. Among the few studies that have investigated the effect of exercise training in older patients with cancer, most have focused on older patients with early-stage breast, prostate and colorectal cancer. Especially, research investigating the effect of exercise training in older patients with advanced cancer is lacking [37].

### Pancreatic, biliary tract and lung cancer

With median ages around 65 to 70 years at diagnosis, pancreatic cancer (PC), biliary tract cancer (BTC) and lung cancer (LC) are malignancies highly associated with aging. These cancers are most often diagnosed at an advanced stage, and therefore the majority of patients are treated with palliative therapies. Focusing on LC, several previous studies have provided evidence that exercise training is feasible, safe and beneficial for patients with LC before, during and after anti-cancer treatment [38–41]. Demonstrated effects include reduced surgical complications, reduced levels of symptoms and side effects, and improvements in physical function and capacity [38–41]. Only one prior exercise-based study has focused specifically on older patients with LC. In this study, Lai et al. [42] investigated the effect of a short-term preoperative pulmonary rehabilitation program in 60 older patients (≥70 years) with non-small cell LC (NSCLC) scheduled to lobectomy, and demonstrated significant improvements in functional capacity and peak expiratory flow, as well as reductions in length of hospital stay and post-operative complications. Exercise training for patients with PC and BTC is a relatively unexplored research field. In a randomized controlled trial, Yeo et al. [43] investigated the effect of a 12-week home-based walking program in 102 patients with resected PC or periampullary cancer. After study completion, patients in the intervention group were walking twice as far as patients in the control group, and had significant reductions in pain and fatigue, as well as improved self-reported physical functioning [43]. In a pilot study from 2014, Jensen et al. [44] investigated the feasibility of exercise training in 26 patients with advanced gastrointestinal cancers undergoing palliative chemotherapy. Patients were randomized to one of two groups; resistance training or aerobic exercise on a bicycle ergometer for 12 weeks. In both groups, a significantly decreased level of fatigue was demonstrated from pre to post-assessment, and sleep quality was also significantly improved. However, only minor improvements were seen in a few domains of QoL measures, and no improvements were demonstrated in nausea/vomiting, constipation, dyspnea or appetite/anorexia [44]. No prior studies have investigated the effect of an exercise program specifically for older patients with PC or BTC.

### Aim

The aim of the current study is to investigate the effect of a 12-week multimodal and exercise-based intervention in older patients newly diagnosed with advanced PC, BTC or LC who are undergoing first-line palliative chemotherapy, immunotherapy or targeted therapy.

### Hypothesis

We hypothesize that with a multimodal and exercise-based intervention, participants will maintain physical function. In addition, we believe that participants in the intervention group will have reductions in symptoms and side effects, improved QoL and psychological wellbeing, limited weight loss and muscle wasting, and increased treatment tolerance, compared to participants in the control group.

## Methods

### Design

**Fig. 1:**
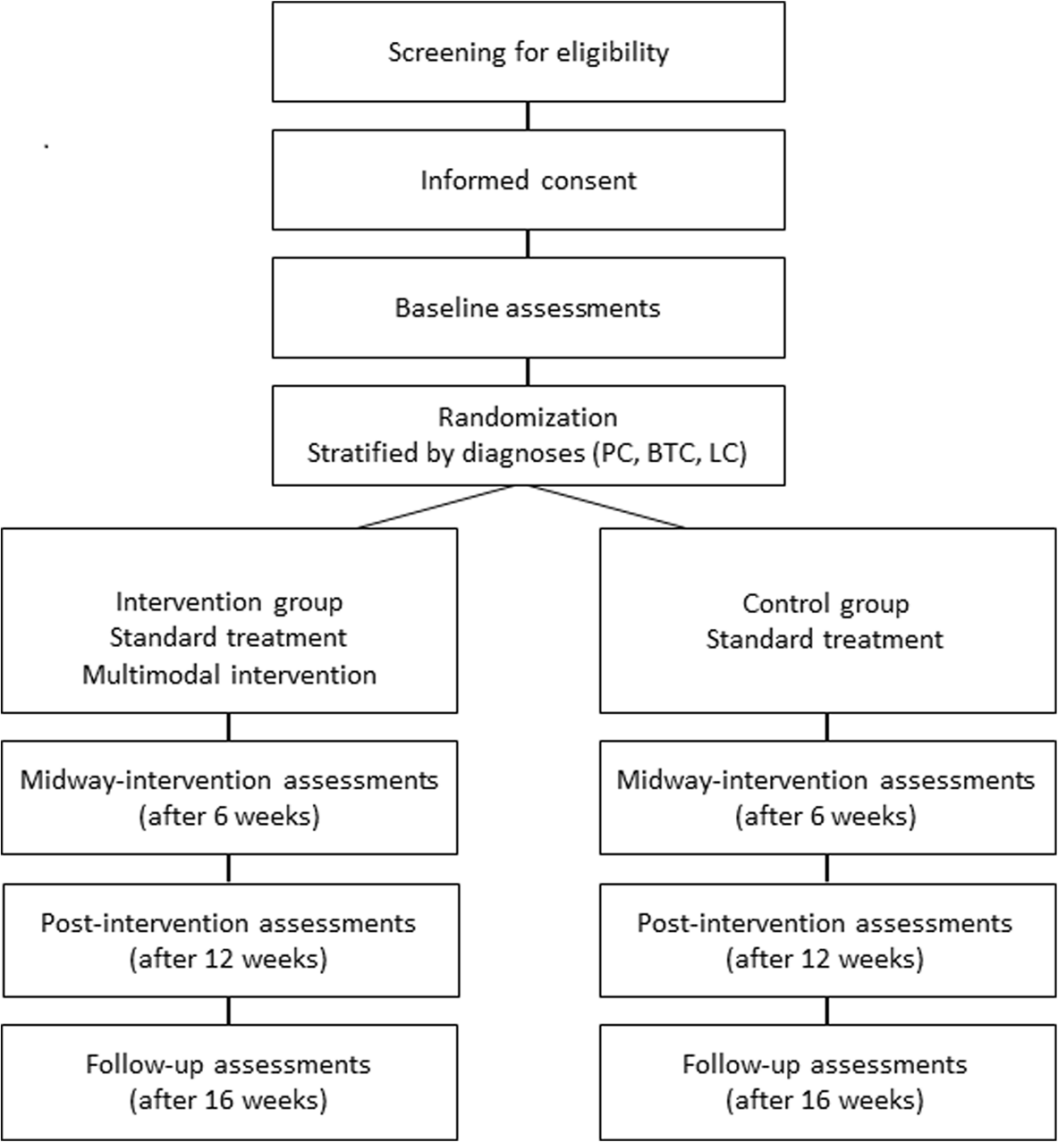
PACE-Mobil-PBL study design. Abbreviations: BTC (biliary tract cancer), LC (lung cancer), PC (pancreatic cancer)

PACE-Mobil-PBL is a prospective two-armed randomized controlled trial. The study is planned to include 100 patients with advanced PC, BTC or LC, who are treated with first-line palliative chemotherapy, immunotherapy or targeted therapy at the Department of Oncology at Copenhagen University Hospital, Herlev and Gentofte, Denmark. The overall study design is illustrated in Fig. 1. This article describes the design of the study and complies with the SPIRIT guidelines for randomized controlled trial protocols, and with the Consensus on Exercise Reporting Template (CERT) recommendations for reporting exercise programs in clinical trials [45].

### Participants

Patients are eligible for inclusion if the following criteria are fulfilled: 1) diagnosis with locally advanced or metastatic PC, BTC or NSCLC within 12 weeks; 2) unresectable cancer; 3) treatment with first-line palliative chemotherapy, immunotherapy or targeted therapy; 4) age ≥ 65 years; 5) Eastern Cooperative Oncology Group (ECOG) performance status score ≤ 2; 6) ability to speak and read Danish, and to provide a signed informed consent form (ICF).

Exclusion criteria are: 1) any physical condition that hinders the execution of physical exercise training; 2) small-cell LC; 3) documented and uncontrolled brain metastases that hinder study participation based on the referring oncologist’s assessment; 4) dementia, psychotic disorders or cognitive diseases or conditions that hinder participation in an exercise-based clinical trial; 5) unstable medical disease or a history of serious or concurrent illness (any medical condition that might be aggravated by exercise training or that cannot be controlled). Patients with documented bone metastases will be excluded from participation if they have a bone metastatic burden or location that poses a risk of pathologic fractures in the performance of exercise training, as assessed by the referring oncologist.

After the initial screening procedure, the research team will obtain ICF and collect all relevant baseline data before randomization.

### Patient involvement

With the aim of involving patients in the research process, individual semi-structured interviews were conducted in older patients with advanced PC, BTC and LC, before the final intervention program was developed. The aim of these pre-interviews was to gain insight into how patients experience and manage symptoms and side effects, to explore their views on exercise training during oncological treatment, and to get their expert advice on the optimal recruitment procedure, intervention composition, and on relevant outcome measures (unpublished results).

### Randomization

After inclusion and baseline assessments, participants are randomly allocated to the intervention group or the control group using a 1:1 block randomization. The block size will only be known by the statistician carrying out the randomization procedure. Randomization will be stratified on diagnosis by using separate randomization lists for each diagnosis group (PC, BTC and LC). The ‘blockrand’ package of the statistical software R was used to generate all randomization lists. Randomization will be administered via the web-based research platform REDCap (Research Electronic Data Capture). Source codes to generate all randomization lists are stored to ensure full reproducibility.

### Intervention

Participants in the intervention group will receive standard oncological treatment and a 12-week multimodal exercise-based intervention comprised of the four following components:

1. *Supervised and group-based exercise training at the hospital setting;* two times a week for sessions of approximately 60 min. The program consists of warm-up exercises of light to moderate intensity, including exercises for balance and flexibility, and a program of progressive resistance training (PRT) that comprises seven resistance training exercises targeting the large muscle groups. In the first two weeks, participants will be introduced to the PRT program, starting with high repetition numbers and low loads. During the intervention period, the volume of the PRT will progress from two to three sets, and the load will increase from 15 to 10 repetitions maximum (yielding a heavier load relative to each participant’s maximum). The progression will be tailored to each individual participant. Three experienced physiotherapists (range 6–17 years) will supervise all training sessions. Training sessions will be performed in groups of up to 12 participants at the rehabilitation facilities at Herlev and Gentofte Hospital, where appropriate strength training equipment is available (Technogym Element series, Gambettola, Italy). For detailed description of the supervised exercise program see Table 1.
2. *Nutritional supplement after supervised exercise training*; to prevent a negative energy balance on training days, a protein supplement (e.g. protein drink or bar, 227–300 cal and 12–20 g protein) will be served for participants after each training session.
3. *An individualized activity program with steps counts, goal-setting and evaluation;* based on each participant’s starting point, preferences and motivation, an individualized activity program will be conducted with the overall aim of maintaining or increasing the level of daily activity. The level of activity will be measured as steps counts with a Garmin Vivofit 3 activity tracker (Garmin, Olathe, Kansas, U.S.). Goal-setting and evaluation in relation to walked steps will be conducted once weekly, face-to-face or by telephone, in collaboration between each participant and a member of the research team.
4. *Motivational and supportive counseling;* participants in the intervention group will participate in two sessions of individualized nurse-led counseling. The first session will be held immediately after study entry, and the second will be conducted after 6 weeks. Counseling will be based on a holistic assessment of each participant’s life situation and potential challenges. Each participant will be asked about potential problems in different life domains (physical, functional, psychological/emotional, social, and existential/spiritual). Based on identified needs, participants will be challenged with questions about the characteristics of the problem(s), factors that may influence the problem(s), and their problem management strategy. The purpose of this process is to activate the participants and to promote self-management. Also, advice and counseling on identified problems will be provided, and followed up if needed. If participants present any complex medical problems, they will be encouraged to contact their oncologist or general practitioner, as appropriate. As mandatory components, all participants will be informed about expected bodily reactions to exercise training, and advised about sufficient nutrition during the intervention. If participants need specialized nutritional counseling, they will be referred to a dietician.

**Table 1:** Description of the supervised exercise program

Duration of exercise program	12 weeks (24 sessions)
Duration of exercise sessions	60 min (10 min of warm-up, 45 min of PRT and 5 min of stretching)
Exercise frequency	2 times a week
Rest period between exercise sessions	2–5 days
Warm-up	Exercises of light to moderate intensity with elements of balance and flexibility training (e.g. walking around in circles with changing directions, walking on toes and heels, floor touch, walking lunges, exercises on balance board)
Description of PRT exercises	Chest press, abdominal crunch, leg press, leg curl (hamstrings), leg extension (quadriceps), lower back and low row
No. of repetitions (PRT)	15 RM (session 1–2), 12 RM (session 3–13), 10 RM (session 14–24)
No. of sets per session (PRT)	2 (session 1–6), 3 (session 7–24)
Rest period between sets (PRT)	60 s

Abbreviations: No. (number), *PRT* progressive resistance training, *RM* repetition maximum

### Controls

Participants in the control group will receive standard oncological treatment. No restrictions on physical activity are made for control participants.

### Study outcome measures

The primary endpoint is the between-group difference in within-group changes in physical capacity and lower extremity strength measured with the 30-s chair stand test (30s-CST) at the post-intervention assessment. Secondary endpoints include feasibility measures, adverse events, and changes in functional performance, muscle strength, body composition, body weight, patient-reported performance status, symptom burden, symptoms of depression and anxiety, QoL, physical activity, treatment tolerance and toxicity, inflammatory biomarkers, hospital admissions and mortality. The outcome measures for both groups are described in the following section.

#### Physical tests

To measure physical function and lower extremity strength, the 30s-CST will be used (primary outcome) [46]. The 30s-CST is considered as highly relevant for older patients with cancer, as the ability to rise from a chair is regarded a prerequisite for functional independence. The 30s-CST is a widely used and validated physical measure, especially among older people, and it has also been used and tested among patients with cancer [47–49]. Physical performance will also be assessed with a 6 and 10-m gait speed test. Gait speed is a suitable measure to use in a clinical rehabilitation setting, and has a documented predictive value for functional and cognitive decline, falls, independence, and mortality [50, 51]. To measure physical capacity and endurance, the six-minute-walk test (6MWT) will be used [46]. The 6MWT measures the distance an individual is able to walk over a total of six minutes on a hard and flat surface. The test was originally developed for use in frail older patients, but has been used and validated in a variety of different populations [52, 53]. To measure physical function and strength of the upper body, the handgrip strength test will be used [54]. Research has shown that handgrip strength may be independently associated with functional capacity, QoL and survival in patients with advanced cancer [55]. Detailed descriptions of the performance of physical tests can be found in an additional file (see Additional file 1).

#### Feasibility measures

Feasibility of the intervention will be evaluated as acceptability, attrition and adherence. Acceptability will be measured as the number of eligible patients who agree to participate in the study. Reasons for declining study participation will be listed and evaluated. Attrition will be registered as the number and percentage of participants who do not complete the study requirements. For each component of the intervention adherence will be assessed as the percentage of study parts completed. Data on adverse events related to the intervention will be monitored according to the National Cancer Institute Common Terminology Criteria for Adverse Events, version 4.0 (NCI CTCAE v4.0).

#### Patient-Reported Outcome Measures (PROM)

Patient-reported performance status (PRPS) will be assessed using the ECOG performance status [56]. To measure symptom burden, the M.D. Anderson Symptom Inventory (MDASI) version 1 will be used [57]. MDASI is a multi-symptom PROM assessing the severity of symptoms and their interference with daily living [57]. To assess QoL, the European Organisation for Research and Treatment of Cancer Quality of Life Questionnaire C30 (EORTC QLQ-C30) version 3.0 will be applied [58]. The EORTC-QLQ-C30 is designed to be cancer specific, and consists of 30 items distributed throughout different functional and symptom scales [58]. To assess symptoms of depression and anxiety, the Hospital Anxiety and Depression Scale (HADS) version 3.0 will be used [59]. HADS comprises 14 items and is designed to measure general depression and anxiety by self-administration [59].

#### Physical activity

Level of physical activity will be measured by step counts using a Garmin Vivofit 3 activity tracker (Garmin, Olathe, Kansas, U.S.). The average number of steps walked measured over three consecutive days will be used as an estimate of physical activity.

#### Qualitative assessment of participants’ experiences

Qualitative semi-structured interviews will be conducted with a sample of participants from the intervention group post intervention to explore participants’ experiences and satisfaction with the intervention.

#### Treatment tolerance and toxicity

Data on dose-reductions and treatment discontinuations will be registered prospectively throughout the intervention period. Toxicity will be registered according to the NCI CTCAE v4.0.

#### Body measures

Weight, height and body mass index (BMI) will be assessed using standard procedures (no shoes, light clothing). Body composition will be assessed by whole-body dual-energy x-ray absorptiometry (DXA) scan (GE lunar iDXA, GE Healthcare Technologies, Madison, Wisconsin, U.S.), and by bioelectrical impedance (BI) assessment (Body Composition Monitor, Fresenius Medical Care, Bad Homburg v.d.H., Germany).

#### Inflammatory biomarkers

To explore the influence of exercise training on inflammation, data on inflammatory biomarkers C-reactive protein (CRP), interleukin 6 (IL-6), and Chitinase-3-like protein 1 (CHI3L1), also known as YKL-40, will be collected from medical records or through cooperative research projects.

#### Hospital admissions

Data on number, causes, and length of hospitalization during the study period will be collected from medical records.

#### Mortality/survival

Incidences of deaths and causes of deaths will be registered throughout the study period by review of medical records.

Frequency and timing of study assessments are shown in Table 2. REDCap will continuously be used for data entry during the study period, and subsequently for data storage.

**Table 2:** Schedule of study assessments

Time (week no.)	-2 to 0	0	1	2	3	4	5	6	7	8	9	10	11	12	13	17
Enrollment																
Eligibility screen	X															
Informed consent	X															
Randomization		X														
Intervention			X---------------------------------------------------------------------------------------------------------------------------X													
Physical tests and examinations																
30s-CST (primary outcome)	X								X						X	X
6MWT	X								X						X	X
Handgrip strength	X								X						X	X
Gait speed test	X								X						X	X
Physical activity^a^	X														X	
DXA	X														X	
BI	X														X	
Body weight^a^	X								X						X	X
Inflammatory biomarkers	X								X						X	X
PROMs																
PRPS	X								X						X	X
MDASI^a^	X								X						X	X
EORTC-QLQ-C30	X								X						X	X
HADS	X								X						X	X
Qualitative exploration																
Individual Interviews															X	
Feasibility																
Adherence	X	X	X	X	X	X	X	X	X	X	X	X	X	X		
Attrition	X	X	X	X	X	X	X	X	X	X	X	X	X	X	X	X
Adverse events	X	X	X	X	X	X	X	X	X	X	X	X	X	X		
Oncological treatment																
Treatment related toxicity^b^	X	X	X	X	X	X	X	X	X	X	X	X	X	X	X	X
Dose-reductions^b^	X	X	X	X	X	X	X	X	X	X	X	X	X	X	X	X
Hospitalizations	X	X	X	X	X	X	X	X	X	X	X	X	X	X	X	X
Survival	X	X	X	X	X	X	X	X	X	X	X	X	X	X	X	X

^a^Once weekly for participants in the intervention group ^b^Registered for every treatment course

Abbreviations: *30s-CST* 30-s chair stand test, *6MWT* six-minute-walk-test, *BI* bioelectrical impedance, *DXA* whole-body dual-energy x-ray absorptiometry, *EORTC-QLQ-C30* European Organisation for Research and Treatment of Cancer Quality of Life Questionnaire C30, *HADS* Hospital Anxiety and Depression Scale, *MDASI* M.D. Anderson Symptom Inventory, *PRPS* patient-reported performance status

### Blinding procedures

Assessments of the primary outcome 30s-CST and all other physical tests will be performed by blinded physiotherapists. Due to the nature of the study, study participants are not blinded.

### Statistics

#### Sample size

No prior studies have investigated the clinically important difference in the 30s-CST in patients with cancer. According to a previous study focusing on patients with osteoarthritis, the clinically important change in the 30s-CST was set at 2.6 repetitions [60]. Based on results from prior studies in patients with advanced cancer, a standard deviation (SD) of approximately 3 has been reported [61, 62]. To be able to detect a between-group difference in the within-group changes of 2.6 repetitions in the 30s-CST, and to obtain a type I error rate of 5% and a power of 90%, a sample size of 29 participants per study arm will be needed. To account for a dropout rate of approximately 40%, it has been decided to increase the group size to 50. Thus, a total of 100 participants will be included in the trial.

#### Analyses

Feasibility measures will be reported as numbers and percentages. Baseline characteristics will be calculated for all participants (total), and separately for the intervention and control group. For all quantitative variables the median number and interquartile range (IQR) will be calculated, and for nominal variables the number and percentage distribution will be calculated. Results from physical tests, blood test, body measures, questionnaires, hospitalizations and toxicity-measures will be reported as means and SDs or as median and IQR, as appropriate. Change over time in ordinal categorical values will be evaluated by a trend test using logistic regression. Within-group and between-group differences in continuous-level data will be performed using independent T-tests. Overall and cancer specific survival will be analyzed with the Kaplan-Meier method, competing risk and Cox regression analyses. The significance level of all tests is set at *p* < 0.05 and analyses will be carried out in R by the program’s statistician (AT).

## Discussion

We have designed a randomized controlled trial to investigate the effect of a multimodal exercise-based intervention for older patients with advanced PC, BTC and LC undergoing first-line palliative treatment. The elements of exercise in the PACE-Mobil-PBL intervention are designed in accordance with Danish and international recommendations for exercise training in healthy individuals and in patients with cancer [63–65]. Focusing on older patients with advanced PC, BTC and LC, several factors have been considered in targeting the intervention, including age-related limitations and comorbidities. Due to the high incidence of cancer cachexia and sarcopenia among these patient groups, the main priority of the exercise program lies on resistance training, whereas high intensity cardiovascular training that requires high energy consumption is avoided. With the aim of avoiding inactivity, an individualized light to moderate intensity home-based activity program based on step counts will be developed for each participant. The multimodal intervention also includes two individualized counseling sessions. The aim of the counseling is to help participants to cope with their current situation in different life domains. The content related to selected domains and questions in the counseling is inspired by the comprehensive geriatric assessment, and by Danish recommendations for the basic palliative assessment [66, 67]. As patients with advanced PC, BTC and LC are at high risk of multiple debilitating symptoms and side effects during oncological treatment, such as fatigue, anorexia, pain, respiratory problems and psychological distress [68–70], counseling sessions will specifically focus on optimizing participants’ symptom management. The approach in guiding participants to manage their symptoms and side effects is inspired by the Integrated Approach to Symptom Management developed by Larson et al. [71]. Information on sufficient nutrition during the course of cancer treatment will be incorporated as a mandatory element in the first counseling session, and a protein supplement will be served to all participants after all training sessions. Focus on nutrition is incorporated as nutritional status is recognized as an independent predictor of cancer-related outcomes such as treatment tolerance and mortality [72, 73]. Furthermore, evidence suggests that malnutrition is highly prevalent in patients with cancer, particularly among older patients with cancer [74].

In the PACE-Mobil-PBL intervention, motivation is considered a key factor in engaging participants in exercise training and symptom management, both short and long term. The approach to participants in the nurse-led counseling is inspired by the concept and techniques from Motivational Interviewing [75]. Motivational Interviewing is a collaborative and person-centered method that helps people to resolve ambivalence, and to strengthen their internal motivation needed for a behavior change. Motivational Interviewing has been applied in various settings, and has proven to be an effective counseling strategy in life style changes among patients with cancer [76]. Goal-setting is incorporated as an element in the home-based activity program, as it is recognized as an effective method to achieve behavioral changes, and to enhance motivation and self-efficacy [77].

With the aging population and expected increase in cancer incidence, especially among older people, new approaches of early rehabilitation are needed in order to maintain physical function, independence and QoL in older patients with advanced cancer. PACE-Mobil-PBL has the potential to become a new model of early rehabilitation for older patients with advanced PC, BTC and LC. In the long term, it is our goal to investigate the multimodal program’s effect among older patients with various malignancies in order to map out the effects, common features and differences across cancers, with an overall focus on older patients with cancer.

## Additional file

Additional file 1: Detailed description of physical tests. A detailed description of the performance of physical tests in the study, including practical conditions, tools and instructions. (DOCX 14 kb)

## Abbreviations

30s-CST: 30-second Chair Stand Test
6MWT: Six-Minute-Walk-Test
ADL: Activities of Daily Living
BI: Bioelectrical Impedance
BMI: Body Mass Index
BTC: Biliary Tract Cancer
CHI3L1 (YKL-40): Chitinase-3-like protein 1
CRP: C-Reactive Protein
DXA: Dual-energy X-ray Absorptiometry
ECOG: Eastern Cooperative Oncology Group
EORTC-QLQ-C30: European Organisation for Research and Treatment of Cancer Quality of Life Questionnaire C30
HADS: Hospital Anxiety and Depression Scale
ICF: Informed Consent Form
IL-6: Interleukin 6
IQR: Interquartile Range
LC: Lung Cancer
MDASI: M.D. Anderson Symptom Inventory
NCI CTCAE v4.0: National Cancer Institute Common Terminology Criteria for Adverse Events, version 4.0
NSCLC: Non-Small Cell Lung Cancer
PC: Pancreatic Cancer
PROM: Patient-Reported Outcome Measure
PRPS: Patient Reported Performance Status
PRT: Progressive Resistance Training
PS: Performance Status
QoL: Quality of Life
REDCap: Research Electronic Data Capture
SD: Standard Deviation
