# Supplementary material for: Engaging the older cancer patient; Patient Activation through Counseling, Exercise and Mobilization – Pancreatic, Biliary tract and Lung cancer (PACE-Mobil-PBL) - study protocol of a randomized controlled trial
Source: BMC Cancer. 2018 Sep 27;18:934. doi: 10.1186/s12885-018-4835-2 (PMC6161425; doi:10.1186/s12885-018-4835-2)
Supplement: Supplementary file 1 — Detailed description of physical tests. A detailed description of the performance of physical tests in the study, including practical conditions, tools and instructions. (DOCX 14 kb) [file 12885_2018_4835_MOESM1_ESM.docx]

**Additional file 1: Detailed description of physical tests**

30-second chair stand test

Using a chair with a seat height of 43-45 cm, participants will be instructed to sit in the middle of the chair with their back straight, arms crossed over the chest, and both feet resting flat of the floor. Participants will be instructed in using the correct technique before the test (both verbally and with demonstration). On the instructor’s signal “ready, set, go”, participants will be asked to stand straight and then return to a seated position as many times as possible in 30 seconds. The number of full stands (full extension of knees and hips) performed in 30 seconds, will be used as the test score. During the test, each stand will be registered using a manual and soundless hand counter, and no cheering will be provided. A modified version of the 30s-CST where participants are allowed to use the armrests, can be used among participants who are not able to rise from a chair without using their arms. If a participant is only able to perform the modified version at one time point, he/she will be asked to perform the same modified test at all following assessments. However, if a participant improves and thus becomes able to perform the standardized test, he/she will be asked first to perform the modified test, and then to perform the standardized test after a 15 minute rest period.

Gait speed test

Usual gait speed will be measured on flat and straight courses of 6 and 10 meters, respectively. The courses will be clearly marked with taped lines. Participants will get verbal instructions on how to perform the test, and it will be emphasized that the test must reflect their usual gait speed, and not their maximum gait speed. Participants will be asked to stand with their feet behind the start line, and then on the instructor’s command “ready, set, go” to walk the course at their usual pace. Timing will be started when the participant’s first foot touches the floor, and will be stopped when the participant’s first foot has completely crossed the finish line. The faster of two attempts for each distance will be used as test scores.

Six-minute-walk-test

Participants will be asked to walk as far as possible in six minutes on a flat 20-meter straight course. Both ends of the course will be clearly marked with cones. The participants will be instructed not to talk during the test unless they feel unwell. The test will be started on the instructors signal “ready, set, go”. Time left of the test accompanied by a motivational phrase such as “you are doing well” will be called out every 60 seconds. The distance walked in meters after one attempt will be used as the test score. If a participant stops to rest during the test, the instructor will keep the stopwatch running, and will encourage the participant to continue as soon as possible.

Handgrip strength test

Handgrip strength will be measured using a hand-held digital Jamar dynamometer. The best of three attempts performed with the dominant hand will be used as test score. Participants will be sitting down during the assessment with both feet on the ground, shoulders adducted and naturally rotated, and the elbow flexed at a 90⁰ angle and supported on the armrest. On the instructors signal “Squeeze.. harder, harder, harder..”, participants will squeeze the dynamometer as long and hard as possible, and will stop on the instructor’s signal “relax”.
